# Comparative Transcriptomic and Lipidomic Analyses of Human Male and Female Meibomian Glands Reveal Common Signature Genes of Meibogenesis

**DOI:** 10.3390/ijms20184539

**Published:** 2019-09-13

**Authors:** Igor A. Butovich, Nita Bhat, Jadwiga C. Wojtowicz

**Affiliations:** Department of Ophthalmology, University of Texas Southwestern Medical Center, Dallas, TX 75390, USA; nbhat@houstonmethodist.org (N.B.); fenotipojw@gmail.com (J.C.W.)

**Keywords:** meibomian glands, meibum, meibogenesis, lipids, genes, mass spectrometry, chromatography

## Abstract

Meibum is a lipid secretion that is produced by holocrine Meibomian glands (MGs). MGs are a specialized type of sebaceous glands that are embedded in the human eyelids. Chemically, meibum and sebum are different. A detailed characterization of lipidome and transcriptome of MG is required to deconvolute a complex and poorly characterized array of biosynthetic reactions (termed *meibogenesis*) that lead to formation of meibum. Changes in the composition and quality of meibum have been linked to various ocular disorders, some of which are more prevalent in males, while others in females. To establish the role of gender in meibogenesis in humans, we characterized MG transcriptomes and lipidomes of females and males, and identified signature genes of meibogenesis in both genders. Specimens of MG tissues were subjected to mRNA microarray analyses. Chemical composition of meibum samples was assessed chromatographically and mass spectrometrically. Both targeted and untargeted approaches were used. About 290 signature genes of meibogenesis were identified. The analyses of their expression patterns demonstrated no major differences between the genders. Lipid profiling of major classes of meibomian lipids, such as wax esters, cholesteryl esters, free cholesterol, (*O*)-acylated omega-hydroxy fatty acids (OAHFA), cholesteryl esters of OAHFA, and triacylglycerols, also demonstrated only minor (and random) differences in these lipids. The results of transcriptomic analyses correlated well with lipidomic data. Taken together, our data imply that in males and females, meibogenesis proceeds in a similar fashion, yielding secretions with similar, highly conserved, compositions. This finding is important for designing novel, gender-independent diagnostic and therapeutic approaches to various MG-related diseases and pathological conditions.

## 1. Introduction

Holocrine Meibomian glands (MGs) that are embedded into tarsal plates (TPs) of eyelids of humans [1] and most mammals are responsible for synthesizing and expressing a lipid-rich secretion termed meibum [2]. A wide-spread pathological condition—MG dysfunction, or MGD [3]—is believed to be one of the major causes of Dry Eye in humans. MGD has been linked to changes in the quality (i.e., chemical composition) of meibum produced by MGs [4,5,6]. While the higher prevalence of Dry Eye in women [7] and MGD in men [8] implies the possibility of sex-related differences in the quality of meibum [9], the role of sex per se in the biosynthesis of meibomian lipids by MGs (termed *meibogenesis* [10,11] for brevity and by analogy with terms such as lipogenesis, steroidogenesis, glycogenesis) remains unclear. Importantly, sex hormones (such as androgens, estrogens, progesterone) have been proposed to regulate the development and functions of MGs in experimental animals and humans [12,13,14,15,16]. However, those efforts to establish the roles of sex hormones in regulation of the biosynthesis of meibum are open to questions because of critical deficiencies in the analytical techniques that have led to the incorrect identification of lipids, as discussed earlier [17,18,19]. Notably, meibomian lipids are very different from products of regular lipogenesis found in other cells, tissues, and organs (except, possibly, the secretions of the related sebaceous glands), indicating that substantial differences in the biosynthetic pathways and regulatory mechanisms may exist between meibogenesis and lipogenesis. Thus, concepts and hypotheses that are applicable to, and well-suited for, other lipid-producing systems, cannot not be automatically applied to MGs.

Recently, we reported the results of a study aimed at the evaluation of the role of sex in meibogenesis in mice, which had been shown to be a close model of meibogenesis in humans [10,20]. Importantly, no noticeable differences in the chemical composition of meibum and in the expression patterns of corresponding genes were found for age- and diet-matched mice: Lipid and gene expression profiles (GEPs) of males and females were remarkably similar to each other [21,22]. It seems that in tested experimental animals, gender alone did not have any measurable impact on the quality of meibum in healthy young adult mice. The amount of meibum produced by males, however, was considerably higher than the meibum production in females, which correlates well with generally larger sizes of the MGs of the former.

Thus, the main goals of this study were to: (1) Characterize human MG transcriptomes and lipidomes in males and females; (2) define signature genes of meibogenesis by correlating GEPs with the lipid profiles in MGs; and (3) determine if there were any major sex-specific differences in the GEPs related to meibogenesis and in the lipid composition of meibum.

## 2. Results

### 2.1. Comparative Lipidomic Analyses of Human Male and Female Meibum

**Figure 1:**
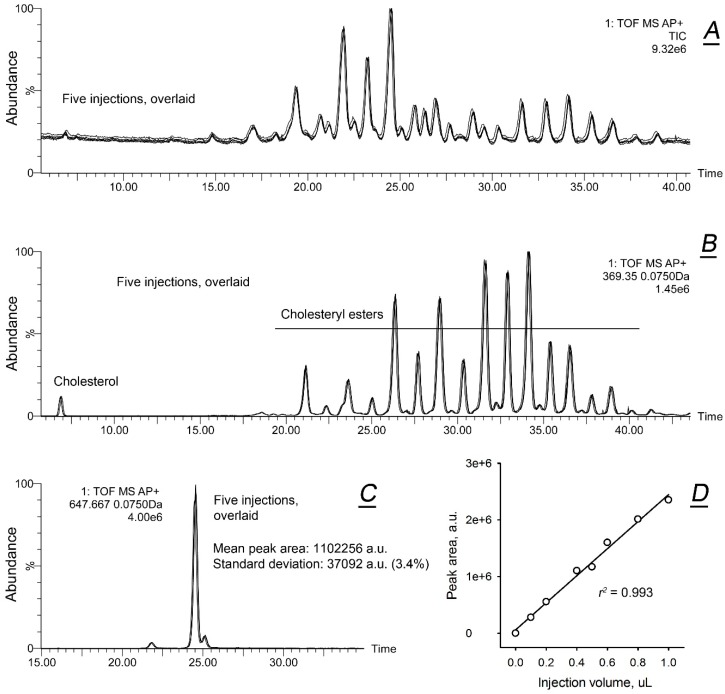
Validation of the reverse phase ultra-high performance chromatographic/mass spectrometric (RP-UPLC/MS) procedures. A representative sample of human meibum was analyzed five times using RP-UPLC/MS. (**A**) Five consecutive injections of a representative sample of human meibum produced identical total ion chromatograms (overlaid). (**B**) Five extracted ion chromatograms of ion *m/z* 369.355, overlaid. The ion is spontaneously produced by free cholesterol and all cholesteryl esters in positive ion mode TOF atmospheric pressure chemical ionization APCI (labeled as AP+) MS experiments. (**C**) Five extracted ion chromatograms of ion *m/z* 647.667, overlaid. The ion is a proton adduct of a wax ester with a molecular formula of C_44_H_86_O_2_. The RP-UPLC/MS peak areas were determined by integrating the chromatographic traces using MassLynx software. (**D**) Evaluation of the linearity of the dose/signal curve produced by injecting various volumes of the same meibum solution. The analyte was the same C_44_H_86_O_2_ meibum wax ester as in (**C**).

First, reproducibility of the reverse phase ultra-high performance chromatographic/mass spectrometric (RP-UPLC/MS) analyses was tested. Five total ion chromatograms (TIC) of a representative meibum sample were obtained in a sequence and plotted together (Figure 1A). The chromatograms were almost identical in terms of the intensities of chromatographic peaks, their number and retention times (RT). Next, chromatograms of free cholesterol (Chl) and cholesteryl esters (CE) were extracted from the same five datasets and superimposed with each other. These five extracted ion chromatograms (EIC) were also found to be almost identical (Figure 1B). To quantify possible spontaneous variations in the experimental results, EIC of a typical meibomian wax ester (WE) with *m/z* value of 647.667 (detected as (M + H)^+^ in positive ion mode (PIM)) were plotted and the peak areas of the main peaks were measured (Figure 1C). In this particular experiment, the mean (M) peak area for the five consecutive analyses and the corresponding standard deviation (SD) were 1,102,256 ± 37,092, or about ±3.4%. The standard error for the same dataset was about 1.5%. Based on these estimates, it was reasonable to assume that any change in the lipid profiles of the study samples that was less than ~3.5% should not be considered as a meaningful difference. Finally, linearity of the instrument’s response was evaluated as shown in Figure 1D. The response was found to be highly linear within the tested range of sample volumes, with an *r*^2^ value of 0.993.

**Figure 2:**
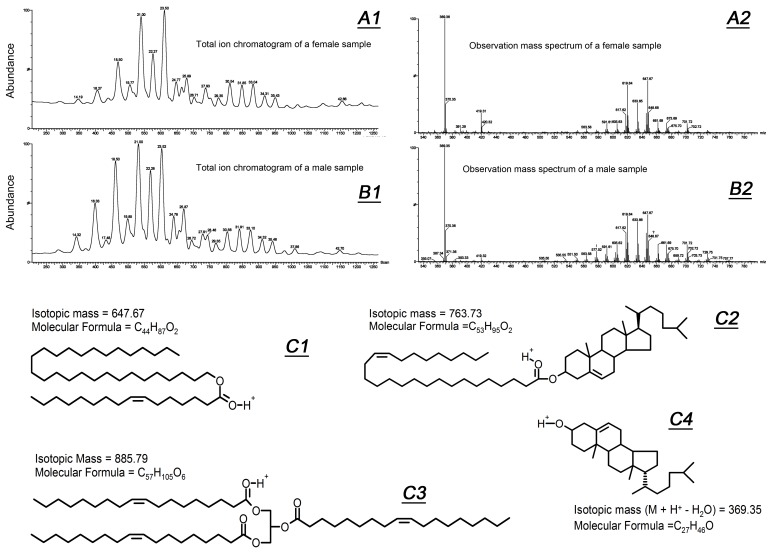
Untargeted lipidomic analysis of female and male meibum samples revealed no major gender-related differences in their chemical composition. (**A1**,**A2**) Total ion RP-UPLC/MS chromatogram of a female meibum sample (**A1**) and an observation mass spectrum of the sample (**A2**). Peak with *m*/*z* 419.32 is produced by diisononylphthalate (a contaminant/plasticizer commonly present in organic solvents). (**B1**,**B2**) Total ion chromatogram of a male meibum sample (**B1**) and its observation mass spectrum of (**B2**). (**C1**–**C4**) Typical representatives of meibomian lipids: Wax ester (**C1**), cholesteryl ester (**C2**), triacylglycerol (**C3**), and free cholesterol (**C4**).

A side-by-side comparison of human male and female meibum specimens was performed similarly to our recent report on mouse male and female TPs [20] and the results of our current RP-UPLC/MS experiments are shown in Figure 2 as representative chromatograms (Figure 2A1,B1) and mass spectra (Figure 2A2,B2). For the reader’s convenience, a few representative meibomian lipids are shown in Figure 2C1–C4.

**Figure 3:**
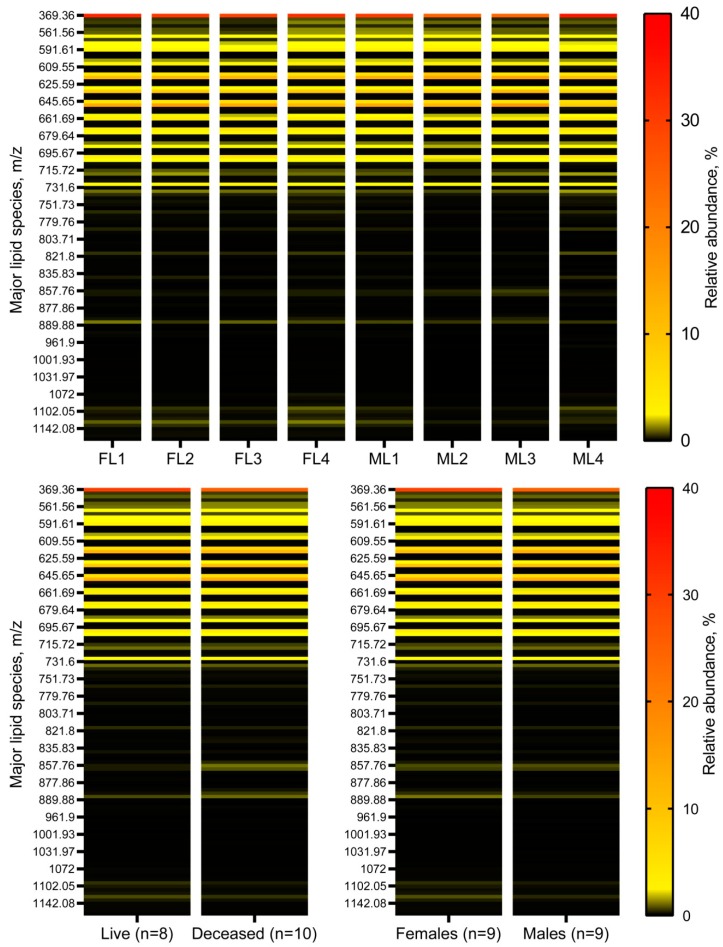
Targeted lipidomic analysis of female and male meibum specimens revealed no major gender-related differences in their chemical composition. (**Upper panel**) Heat maps of meibomian lipids observed in representative female (FL1–FL4) and male (ML1–ML4) samples. Note that due to space constraints only some of the 125 analyzed major lipids are labeled. (**Left lower panel**) Heat maps of averaged meibum samples of live (*n* = 8) and deceased (*n* = 10) donors. No major differences were observed except for somewhat increased levels of normal triacylglycerols (such as those with *m/z* values of 857.76, 859.77 and others) in the deceased group of samples. (**Right lower panel**) Heat maps of averaged meibum samples of female (*n* = 9) and male (*n* = 9) donors. No major differences were noted, with the exception of a slightly elevated (by ~18%) total CEs-to-total WEs ratio and apparently random changes in all groups of lipids.

As the number of lipid species in meibum samples was very high (several hundred lipids, by various estimates), heat maps were chosen to illustrate and present the data. Heat maps of the 125 most prominent lipid analytes detectable in PIM are shown in Figure 3A–C. Due to space constraints, only some of those lipids are labeled on the heat maps. A full description of detected lipids will be reported separately. The makeup of all the major lipid classes of human meibum—WEs, CEs, Chl, cholesteryl esters of (*O*)-acylated ω-hydroxy fatty acids (Chl-OAHFAs) diacylated α,ω-diols (DiADs), etc.—some of which are shown in Figure 2C1–C4, did not vary between the donors: The samples were almost identical with inter-sample differences in individual analytes not exceeding 5–20% of the corresponding means. No statistically significant differences between live and deceased donors were detected, except for a slightly elevated (by ~18%, *p* < 0.05) ratio of total CEs to total WEs in females compared to males. The largest differences in the relative amounts of individual lipids were observed for triacylglycerols (TAGs; *m/z* range 850–900). Considering that TAGs are exceedingly minor components of human meibum (<1% of the total lipid pool, *w/w*), the overall lipid composition of meibum was deemed to be virtually donor-independent regardless the type of the donor.

**Figure 4:**
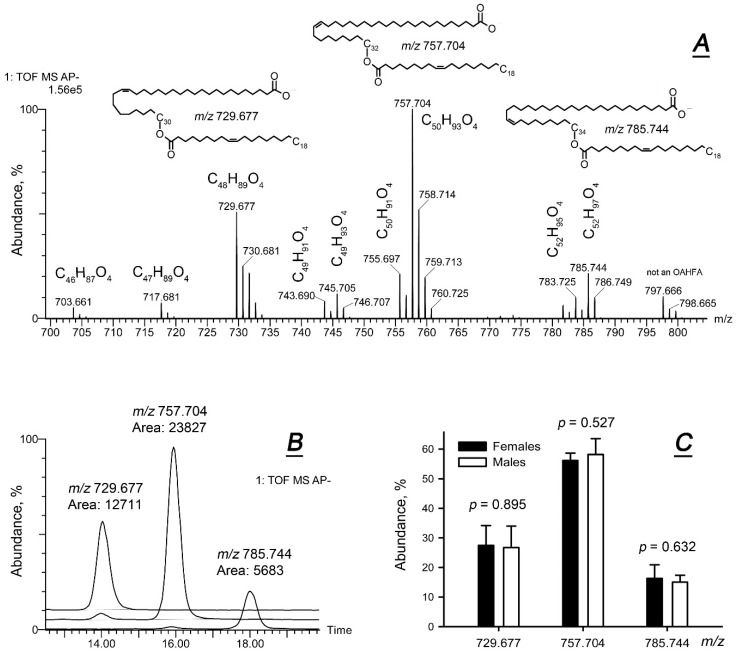
Targeted lipidomic analysis of amphiphilic lipids in female and male meibum. The makeup of (*O*)-acylated omega-hydroxy fatty acids (OAHFAs) showed no differences between the sexes. (**A**) An observation spectrum of a representative human meibum sample obtained in negative ion mode and structures of three prominent OAHFA species. (**B**) Extracted ion chromatograms of three major OAHFAs of human meibum, overlaid and integrated. (**C**) Relative abundances of three representative OAHFA species in female and male meibum (*n* = 9 for each gender group). No differences were observed for other OAHFA species, either.

Amphiphilic lipids, such as (*O*)-acylated ω-hydroxy fatty acids (OAHFAs)—an important and the largest class of such lipids in meibum—are poorly detectable in PIM. Therefore, they were studied in negative ion mode (NIM) (Figure 4). A typical observation NIM mass spectrum of a sample is shown in Figure 4A. The high resolution spectra allowed for a reliable determination of molecular formulas of corresponding compounds, while integration of their EIC made it possible to estimate their apparent abundances within each study sample (Figure 4B; three major species of OAHFAs are shown). The analyses revealed no statistically significant differences between males and females (Figure 4C).

### 2.2. Untargeted Transcriptomic Analyses of Male and Female Tarsal Plates

**Figure 5:**
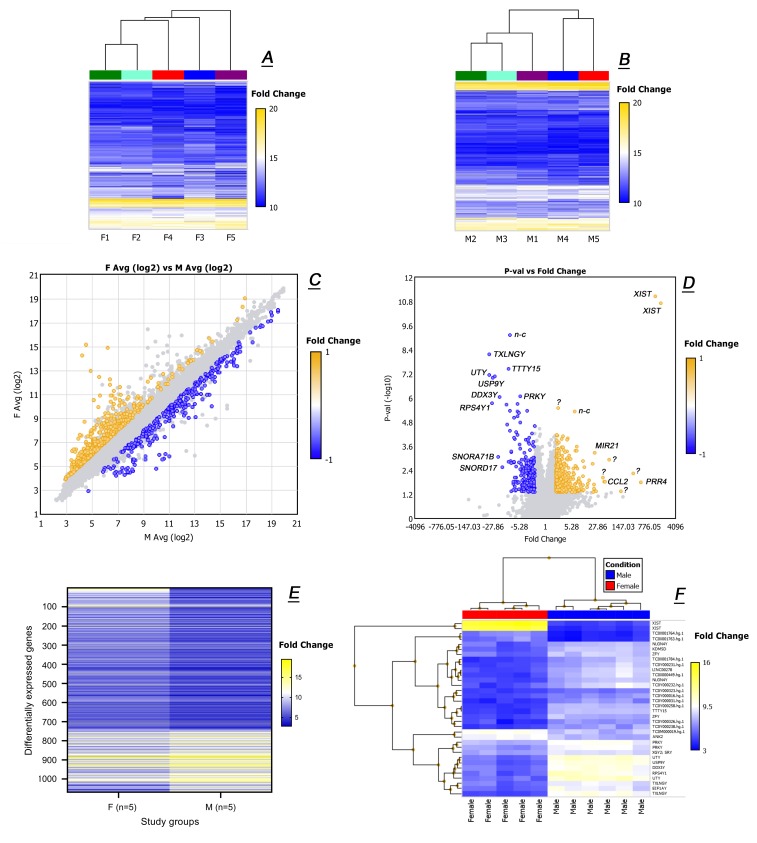
Untargeted transcriptomic analyses of female and male tarsal plates revealed no major intra and inter-group differences in their gene expression patterns except for known gender-specific markers. (**A**) Heat maps of GEP in five female samples F1–F5. (**B**) Heat maps of GEP in five male samples M1–M5. (**C**) A Log2 scatter plot of all genes detected in averaged male (*n* = 5; M) and female (*n* = 5; F) tarsal plate specimens. (**D**) A “volcano” plot of averaged male (*n* = 5) and female (*n* = 5) tarsal plate specimens. Note that some genes were of non-protein coding nature (labeled as *n-c*). Unknowns are labeled with a question mark (?). Genes with the highest linear fold change and lowest *p*-value were those with known gender-specific expression patters and no known association with lipid metabolism, except for *ACADVL* and *SMDP3*. (**E**) Side-by-side comparison of more than a thousand genes that are differentially expressed in female (F) and male (M) tarsal plates. (**F**) Heat maps of female and male tarsal plate genes with the highest LFC values.

The next parameter to evaluate was the inter-donor variability of GEP within male and female groups. As plotting all of more than 67,000 detected transcripts was impossible, filtering was applied to analyze only the genes with Log_2_ expression values (Log2) of ≥10. Figure 5 illustrates the results of hierarchical clustering of 2806 (for females, Figure 5A) and 3146 (for males, Figure 5B) genes that met the criteria. This unbiased approach revealed reproducible GEP within male and female groups of donors with very low inter-donor variability.

Then, all female and male TP samples were assembled in two groups according to their respective sexes, and all genes were plotted as shown in Figure 5C. Differentially expressed genes with a Female-to-Male (F/M) linear fold change (LFC) of ≥ (+2) or ≤ (−2) and *p* < 0.05 are shown in color, while those that did not fit the criteria are shown in gray. Among all 67,528 analyzed genes (44,699 protein-coding and 22,829 non-coding transcripts) there were only 1068 genes that passed the filter criteria. Of those that passed, 744 genes were up-regulated, while 324 were down-regulated.

A high correlation coefficient *r*^2^ of 0.943 was calculated for the entire male and female sets of 44,699 protein-coding genes. Expectedly, known sex-specific transcripts, such as *XIST*, *DDX3Y*, *UTY*, *KDM5D*, and others clearly produced sex-specific patterns: F/M Log2 ratios were (15.2/4.5) for *XIST*, (6.0/10.2) for *DDX3Y*, (5.8/11.0) for *UTY*, and (5.6/8.2) for *KDM5D*. This conclusion was verified by comparing the GEP of several known house-keeping and reference genes [23] which were found to be expressed identically gender-wise: *EMC7* (7.7/7.9), *GPI1* (9.6/9.9), *PSMB2* (6.3/6.4), *RAB7A* (14.5/14.5), *REEP5* (9.9/10.0), *SNRD3* (8.1/8.3), *VCP* (8.6/8.8), and *VPS29* (7.9/8.1) with an average SD of 0.3.

A corresponding “volcano plot” is shown in Figure 5D. Only a handful of genes were identified as being expressed highly differentially *and* having highly significant *p*-values, none of which encoded any known enzymes involved in metabolism and storage of lipids in general, and meibogenesis specifically [10,11], except for *ACADVL* and *SMDP3* with F/M LFC of (−2.9) and a borderline (+2.1), correspondingly. When pooled according to the gender and compared side-by-side (Figure 5E), female and male samples looked very similar, except for a small number of genes known for their sex-specific expression patterns (Figure 5F).

### 2.3. Comparative Transcriptomic Analyses of Meibomian Glands and Tarsal Plate Connective Tissues

**Figure 6:**
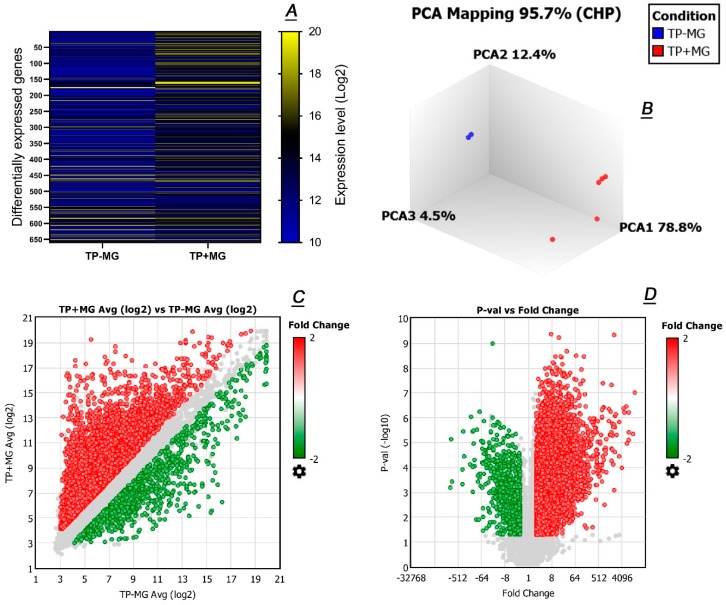
Comparative transcriptomic analyses of human tarsal plates with and without embedded Meibomian glands. (**A**) Averaged heat maps of 650 differentially expressed genes (LFC ≥ (+10) or ≤ (−10)) in male tarsal plates without (TP − MG; *n* = 2) and with (TP + MG; *n* = 5) Meibomian glands. (**B**) Principal component analysis of (TP − MG) and (TP + MG) samples revealed clear grouping of genes based on the types of the specimens. PCA1 was the major contributor to the effect. (**C**) A Log2 scatter plot of all genes detected in (TP − MG) and (TP + MG) samples illustrates extremely high differences between the two groups of samples. (**D**) A “volcano” plot of averaged (TP − MG) and (TP + MG) samples. The differences between the samples were found to be highly significant.

As the number of candidate genes for studying meibogenesis was still very high, we attempted to reduce the number of possible candidates by conducting untargeted differential analyses of GEP of TP specimens with (TP + MG) and without (TP − MG) glands (Figure 6). This approach was to differentiate the genes that were associated with MG per se from the genes that were common in, and coming from, the surrounding connective tissue (CT). Indeed, a heat map diagram (Figure 6A) of 5 male (TP + MG) and 2 male (TP − MG) samples revealed that these two types of tissues were significantly different from each other. Note that only a subset of genes (about 650 entries) with LFCs of ≥ (+10) or ≤ (−10) is shown for clarity. Also noticed were rather high intra-group similarities between the samples, which formed two distinctive groups. The results of principle component analysis (PCA) of the study samples confirmed this conclusion (Figure 6B). A further confirmation of the nonequivalence of (TP + MG) and (TP − MG) samples came from a strikingly different scatter plot with 24,497 (or 36.28% of total) genes being differentially expressed (Figure 6C). Of those 24,497 genes, 23,339 were up-regulated in (TP + MG) samples, and only 1158 were down-regulated. Finally, the “volcano plot” (Figure 6D) was used to estimate reliability of the observed changes, in relation to their magnitudes. The analysis of the plot demonstrated that the differences between (TP + MG) and (TP − MG) samples were large and statistically significant (also compare Figure 6 to Figure 5). Therefore, a conclusion was made that the vast majority of highly expressed genes that were detected in (TP + MG) samples originated from MGs themselves.

### 2.4. Targeted Transcriptomic Analyses of Male and Female Tarsal Plates

Yet, the number of candidate genes was still unmanageably high for further analyses of male vs. female differences and/or similarities. Therefore, a list of about 400 top lipid-metabolism related genes with moderate to high expression levels in TP was assembled using our GEP data and various publicly available databases such as GeneCards, GEO Profiles, SwissProt, BRENDA, KEGG, and others.

Using analytical features of several available on-line search/analysis engines to process the data proved to be rather unproductive because of a very unique combination of genes, enzymes, and their corresponding lipid products in MGs that were clearly different from other human and animal organs, tissues, and cells [10,20]. The uniqueness of MGs is not incorporated into the computer-assisted analytical tools available today, which require manual filtering of the data and correlating GEP with substrate and product specificities of relevant enzymes. A concise summary of our findings is presented below. The results are shown as ratios of mean values determined for females and males (F and M). An average SD for all these measurements was 0.4.

A special consideration was given to genes that were proposed to be involved in the regulation of the physiological functions of MGs—androgen, estrogen, and progesterone receptors ([13,24,25,26] and references cited therein). We have confirmed their expression in human MGs, albeit at predominantly low to moderate levels and with no sex-specific patterns observed. The genes were expressed on a Log2 scale as follows—androgen receptor, *AR* (F/M, 7.1/6.3); estrogen receptor binding site associated antigen 9, *EBAG9* (8.1/8.2); estrogen receptor 1, *ESR1* (3 transcripts, 5.9/5.5, 4.9/4.6, and 6.3/5.9); estrogen receptor 2, *ESR2* (5.1/5.0); G-protein coupled estrogen receptor 1, *GPER1* (5.7/5.5); progesterone receptor, *PGR* (3 transcripts; 5.2/4.9, 5.2/5.0, and 5.6/5.4); progesterone receptor membrane component 1, *PGRMC1* (13.0/12.5); progesterone receptor membrane component 2, *PGMRC2* (9.3/9.3); and progesterone immunomodulatory binding factor 1, *PIBF1* (9.2/9.4).

Next, GEP of genes that encode enzymes of steroidogenesis were evaluated. Some of the key genes of steroidogenesis [27] that were detected in human TP are as follows—*CYP11B1* (4.7/4.7), *CYP11B2* (4.8/4.6), *CYP17A1* (4.8/4.5), *CYP19A1* (4.7/4.5), *CYP21A2* (5.3/5.1), *HSD3B2* (5.6/5.3), *HSD17B3* (4.7/5.1), and *SRD5A2* (5.2/4.7). As with genes described in the previous paragraph, their levels of expression were found to be sex-independent and quite low.

Genes that are involved in biosynthesis of specifically Chl and CEs, on the other hand, were found to be highly expressed in human TPs. These genes include, among others, *ACAT1* (2 transcripts, 8.7/9.1 and 8.0/8.1), *ACAT2* (15.5/15.1 and 13.6/13.3), *DHCR7* (14.7/13.5), *DHCR24* (17.3/16.8), *FDFT1* (9.6/9.9), *HMGCR* (3 transcripts, 17.1/16.9, 14.2/14.8, and 4.1/3.7), *HMGCS1* (16.8/16.5), *HMGCS2* (9.8/10.4), *LCAT* (7.7/7.7), *LSS* (8.1/7.5), *MSMO1* (16.6/16.2), *SOAT1* (17.2/17.5), *SOAT2* (5.1/4.9), and *SQLE* (13.5/12.9). Note that *DHCR7* was the only gene whose Log2 expression values in female samples exceeded that of males by a factor of ~2.

As biosynthesis of CE—a prominent group of lipids in meibum [2,17,28] requires a very extremely long chain fatty acids (VLCFA and ELCFA), the main genes that are involved in FA elongation were quantitated. Those key genes include, among others, a main enzymatic complex that biosynthesizes short to medium chain FA, *FASN* (11.9/11.1) and seven elongases of VLCFA and ELCFA (*ELOVL1* through *ELOVL7*), that are central for FA elongation beyond C_20_ [29,30,31]. These genes produced the following largely sex-independent patterns: *ELOVL1* (12.0/11.7), *ELOVL2* (5.7/5.2), *ELOVL3* (14.6/14.8), *ELOVL4* (18.7/17.6), *ELOVL5* (9.8/9.2), *ELOVL6* (14.4/14.4), and *ELOVL7* (9.0/8.6) with the exception of *ELOVL4* whose Log2 values in females and males differ by a factor of ~2. Note that *ELOVL3* and *ELOVL4* have previously been detected in MGs using immunohistochemical approaches [10].

As FA of the same length (e.g., C_16_, C_18_ C_20_ etc.) can exist as saturated, mono-unsaturated, and polyunsaturated species, it was important to estimate the presence of the genes that regulate the balance of saturated and unsaturated compounds in MGs of females and males. The genes *FADS2* (9.3/8.9), *FADS3* (7.3/6.7), *FADS6* (6.5/6.3), delta-9 desaturase SCD (19.4/19.2), and *SCD5* (also a delta-9 desaturase, 6.8/6.5) were detected in female and male specimens, with *SCD* being on top of the expression list of all enzymes of meibogenesis. This group of genes and corresponding enzymes work together with the *ELOVL*/ELOVL family as the substrate and product specificity of the latter depend on the length and degree of unsaturation of FAs produced by the *FADS*/FADS and *SCD*/SCD families.

Also important for meibogenesis is the process of FA branching as a large portion of meibomian lipids are based on branched FA and/or fatty alcohols (FAl) [10,21,32]. This group of genes include *ACADSB* (8.8/9.0), *BCAT2* (7.4/7.1 and 7.2/7.0), *BCKDHA* (7.4/7.2), *BCKDHB* (10.1/10.0), *DBT* (11.2/11.3), and *ECHDC1* (12.5/12.2).

Another group of key genes, such as *AGPAT5* (9.0/9.4), *CES1* (13.4/14.4), *DGAT1* (7.3/7.1), *DGAT2* (14.0/13.0), *GPAM* (11.1/10.6), *GPAT4* (8.2/8.3), *MGAT2* (7.8/7.2), *MGAT5* (8.6/8.6), *MGLL* (11.0/10.2), and, present in very low levels, *PNLIP* (4.9/4.6), encode enzymes that regulate homeostasis of acylglycerols in general and TAGs specifically via Kennedy and monoacylglycerol pathways (http://www.lipidhome.co.uk/lipids/simple/tag2/index.htm) in a seemingly sex-independent fashion, with the exception of *CES1* and *DGAT2* whose F/M Log2 values differ by a factor of 2.

To synthesize the largest group of meibomian lipids—WE, critically required are genes *FAR1* (10.6/10.6) and *FAR2* (18.0/17.1), which encode fatty acyl-CoA reductases 1 and 2, and *AWAT1* (15.0/14.3) and *AWAT2* (16.7/16.5), which encode acyl-CoA wax alcohol acyltransferases 1 and 2. As expected, these genes were expressed in TPs of all subjects at extremely high levels.

The expression levels of genes that encode proteins that are responsible for lipid storage were also evaluated. Those included, among others, *PLIN1* (7.0/7.0), *PLIN2* (13.4/14.5 and 9.6/9.7), *PLIN3* (9.0/8.1), *PLIN4* (8.4/8.0), and *PLIN5* (7.6/7.1 and 7.0/6.5).

To confirm that these observations were primarily related to MGs themselves, and not the connective tissue, the next step in our analyses was the calculation of LFCs in the levels of expression of most prominent lipid-related genes in MGs of humans in comparison to the CT in tarsal plates (MG/CT). The results below are presented as {*Gene*, (LFC of MG/CT)}. The genes from the steroid receptors and steroidogenesis groups were the least differentially expressed ones among all analyzed groups of genes: *AR* (LFC of 4.3), *ESR1* (3.1), *ESR2* (2.0), *GPER1* (2.6), *EBAG9* (1.4), *PGRMC1* (150.0), *PGRMC2* (1.1), *PGR* (1.4), *PIBF1* (4.0), *CYP11B1* (2.1), *CYP11B2* (2.0), *CYP17A1* (1.6), *CYP19A1* (1.1), *CYP21A2* (1.8), *HSD3B2* (2.1), *HSD17B3* (1.9), and *SRD5A2* (2.0). A clear outlier in this group was the *PGRMC1* gene whose expression level in MGs was more than two orders of magnitude higher than that in CT, implying its possible, but not yet identified, role in the physiology of MGs and meibocytes in general, or meibogenesis specifically.

Metabolism of Chl and CEs in MGs, on the other hand, was extremely high compared to CT with the following LFCs: *ACAT1* (2.9), *ACAT2* (521.5), *DHCR7* (234.0), *DHCR24* (30.3), *FDFT1* (6.6), *HMGCR* (500.4), *HMGCS1* (1952.9), *HMGCS2* (82.1), *LCAT* (3.3), *LSS* (5.7), *MSMO1* (996.6), *SOAT1* (1710.2), *SOAT2* (1.9), and *SQLE* (214.0).

Biosynthesis, desaturation, elongation, and branching of FAs and FAls were also highly up-regulated in the (TP + MG) tissue samples: *FASN* (37.0), *FADS1* (4.0), *FADS2* (27.3), *SCD* (17185.2), *SCD5* (1.2), *ELOVL1* (59.9), *ELOVL2* (1.9), *ELOVL3* (2180.6), *ELOVL4* (14131.9), *ELOVL5* (4.0), *ELOVL6* (609.7), *ELOVL7* (12.6), *ACADSB* (6.8), *BCAT2* (6.4), *BCKDHA* (5.3), *BCKDHB* (32.8), *DBT* (79.2), and *ECHDC1* (103.9).

Since WE are the major group of meibomian lipids, it was not surprising to see four major genes that are essential for biosynthesis of waxes to be elevated in MG: *FAR1* (6.8), *FAR2* (9020.7), *AWAT1* (1026.2), and *AWAT2* (9194.0).

Finally, the *PLIN1*-*PLIN5* genes that encode the PLIN family of proteins (which have been implicated in forming protein coating around lipid droplets in lipid-enriched cells [33]) were also somewhat elevated in MG: *PLIN1* (1.5), *PLIN2* (4.0), *PLIN3* (4.1), *PLIN4* (5.9), and *PLIN5* (5.0).

**Figure 7:**
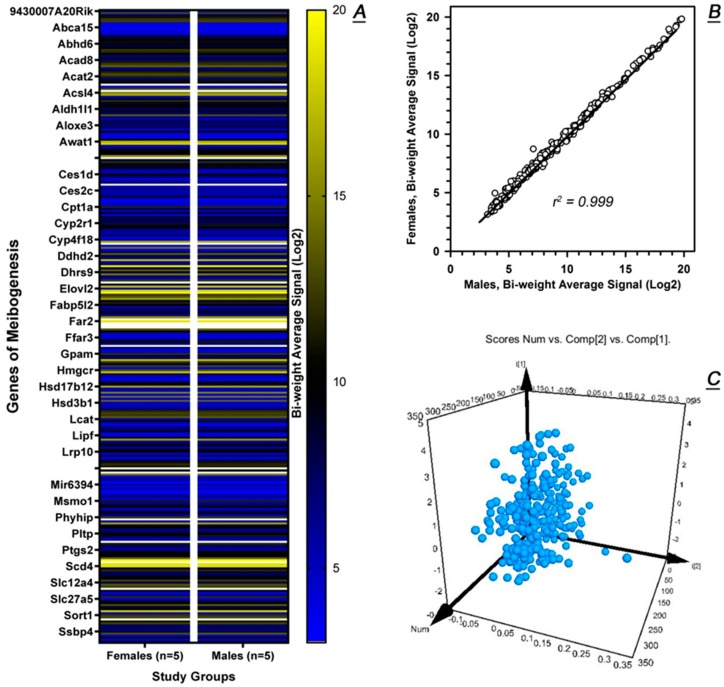
Targeted evaluation of the expression levels of 292 genes of meibogenesis in male and female tarsal plates (*n* = 5 each gender, averaged). (**A**) Heat maps of the genes. Note the extremely high similarities between the genders. (**B**) A Log2 scatter plot of the gene expression levels in males and females. (**C**) (PCA) analysis of the gene expression data demonstrated tight clustering of the genes of meibogenesis, which indicates close similarities in the gene expression profile (GEP) of individual samples from both genders.

This approach allowed us to create a heat map of about 290 signature genes of meibogenesis and conclude that their expression levels in males and females were almost indistinguishable (Figure 7A). The top 50 signature genes from that list are shown in Table 1. A simple correlation analysis (Figure 7B) produced a correlation coefficient *r*^2^ ≥ 0.999, confirming that very little (if any) differences exist in the expression levels of meibogenesis-related genes of males and females. This was further confirmed by a very tight grouping of the signature genes on a PCA plot (Figure 7C).

**Figure 8:**
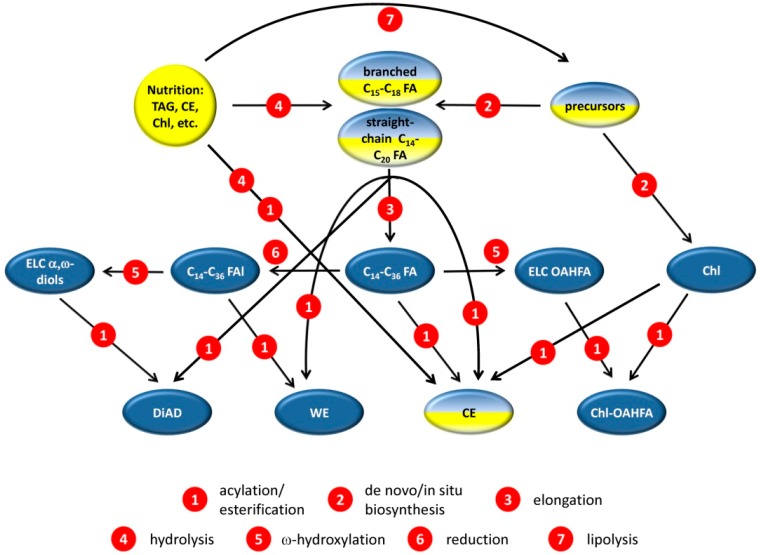
Key steps of meibogenesis. In situ/de novo synthesized lipids are shown in blue, exogenously supplied lipids are shown in yellow; dual coloration indicates a possible mix of exogenously supplied lipids and those that are biosynthesized in meibocytes. Various enzymatic steps are shown in red. Some of the genes and proteins that may be responsible for these steps are listed and discussed in Section 2.4—Results and Section 3—Discussion.

A diagram that summarizes our observations is shown in Figure 8. Note that due to the lack of space, only key enzymatic steps of meibogenesis and their corresponding products are illustrated.

## 3. Discussion

The lipid composition of normal human and animal meibum has been evaluated in a number of earlier studies, the results of which are summarized in several review papers [11,17,19]. Based on comparative transcriptomic and lipidomic analyses of human and mouse TPs, we introduced a concept of meibogenesis, which has been defined as a network of biosynthetic reactions and corresponding regulatory and signaling mechanisms that lead to the formation of meibum [10,11,21,22]. However, its inter-sex similarities and differences remain an understudied topic. In our current study, we have found that the female and male transcriptomes and lipidomes share high similarity with each other when compared side-by-side.

It is important to understand that our comparative analyses of GEP are based on the default (and industry standard) filter criteria: (1) (+2) < LFC < (−2), and (2) ANOVA *p*-value (condition pair) < 0.05. A tighter LFC of > (+1.2) and < (−1.2) (as suggested recently [34]) was also tested, but was deemed to be impractical and misleading because such a stringent LFC criterion could not produce scientifically meaningful results with the given number of samples (below 10 subjects per group). To test these conditions, built-in statistical routines of SigmaStat software package (v.3.5, from Systat Software, Inc.) were used. Indeed, with an α of 0.05, expected SD of 0.3–0.4 in targeted (and ~0.6 in untargeted, i.e., global) experiments, and expected differences in Means of LFC of 0.2, not enough statistical power could be achieved with a typical number of samples per group of 5 to 10 and a target *p*-value of < 0.05: The group sizes must be between 50 and 200 samples per group, which could not be realistically acquired for this kind of experiments. Also, it is highly questionable if such a small difference in LFC could be of any physiological significance as an average SD for all genes with Log2 expression values of ≥ 10 was 0.63 for females and 0.47 for males. However, if one applies the following criteria (which we used for targeted gene expression profiling)—expected difference in Means of LFC of 1 and expected SD between 0.3 and 0.4—the desired power of 0.9 can be achieved with as few as 5 samples per group. Thus, one can conclude that under these conditions there were no statistically significant differences in GEP between males and females. These considerations are essential for understanding the effects (or lack thereof) of sex on meibogenesis.

In our experiments, the expression levels of *ELOVLs*, *SCD*, *FARs*, *DHCRs*, *SOATs*, *AWATs*, *HMGCSs*, *HMGCR*, *ACATs*, *DGATs*, *FASN*, *MSMO1*, and *PLINs*, among others, were extremely high, and demonstrated no statistically significant differences between males and females. Therefore, they were considered to be signature genes of meibogenesis. The expression levels of major housekeeping and reference genes were also statistically indistinguishable. Characteristically, no major differences were observed in the lipid profiles of males and females either (Figure 1), with the major lipid species deviating from their corresponding means by 5–15%, on average. A somewhat elevated ratio of CE to WE in females compared to males was statistically significant, but not high (an increase of ~18%). Currently, it remains unclear if such a difference can have an impact on the ocular surface physiology.

Physiological functions of proteins that these genes encode are essential for biosynthesis of meibomian lipids. As we discussed before [10,11,17,18,19,21,22], and (references cited therein), normal meibum is enriched with lipids with unique features, among which are: (1) The extraordinary length of their FAs and FAls; (2) extensive iso- and anteiso-branching of many FAs and FAls; (3) dominance of FAs and FAls with very few double bonds (typically just one or two), or with no double bonds at all (i.e., saturated ones), and a low (but still measurable) presence of lipids with higher degree of unsaturation [35]; (4) presence of a diverse group of rather unusual (*O*)-acylated ω-hydroxy-FAs (OAHFAs), their cholesteryl esters (Chl-OAHFAs), diacylated α,ω-diols (DiADs), and other less known compounds; (5) high enrichment of meibum with very hydrophobic species such as WEs and CEs (which, together, account for more than 70% of meibum [17]); and (6) an almost complete lack of TAGs, ceramides, phospholipids, sphingomyelins and other lipids that are normal components of other human tissues, and some other distinctive features.

Importantly, many of the signature genes of meibogenesis with known functions (Table 1 and Section 2.4—Results) encode enzymes and proteins that are involved in FA elongation, desaturation, ω-oxidation, FA reduction to FAl, esterification (to make WEs, CEs, OAHFAs, Chl-OAHFAs, and DiADs), while the others are involved in steroidogenesis per se (to make Chl), regular lipogenesis (such as a complex of enzymes FASN), lipid storage (such as PLINs), and transport. A comprehensive discussion of these processes goes far beyond the scope of this manuscript and will be addressed in future studies and publications. However, it is important to note that almost non-existent differences in lipid profiles of meibum and GEP of the signature genes in TP of women and men resemble our recent findings with regard to male and female mice [21] in every detail, and accentuate the fact that meibogenesis is an important and extremely tightly controlled metabolic process that is well protected from rapid changes. Otherwise, larger fluctuations in the lipid profiles and GEP would have been observed in our experiments, which was not the case. Importantly, our experiments with laboratory animals [21] were conducted with young adult mice, while our human lipidomic and transcriptomic studies described in this paper were conducted on an older group of human population in their sixties and seventies. Still, no measurable effect of gender was observed in both studies. This could mean that the effect of aging and/or hormonal status is smaller than one would expect, and additional experiments are needed to address this important matter.

The genes and the proteins discussed above represent only the main enzymatic machinery of the lipid biosynthesis in MG, i.e., the backbone of meibogenesis, and do not concern the mechanisms of its regulation, which undoubtedly involve a host of yet to be identified regulatory proteins and signaling factors. Illuminating in this respect is the lack of any direct evidence of induction of meibogenesis in MG-derived cell cultures by a variety of factors [36,37,38] as no production of meibomian-type lipids in those conditions have been reported to date. However, our data on the GEP of the major genes involved in steroidogenesis and functioning of androgen, progesterone, and estrogen receptors demonstrated that tested genes were expressed almost identically in both genders, and predominantly at low to moderate levels, raising some doubts about their direct involvement in regulation of meibogenesis.

Our new data correlate well with our recent report on lipidomes and transcriptomes of male and female mouse TPs [21] where no measurable differences between the sexes were observed either. Taken together, our data imply that in normal subjects of both genders, meibogenesis proceeds in a similar fashion yielding secretions with similar, highly conserved, gender-independent composition. Importantly, we have observed a large, statistically significant inter-sex difference in the overall quantity of meibum that was produced by MGs of mice [21]: Male mice produced almost twice as much meibum as females, which correlates well with the larger physical sizes of MGs and TPs of the formers. It seems that this difference may, in part, contribute to higher prevalence of Dry Eye in women as a partial loss of the ability of MGs to produce meibum (for example, because of aging or hormonal imbalance) would have a greater impact on the ocular surface of females than males, as both genders have eyes of identical physical dimensions [39].

In general, the use of tissues introduces a possibility of errors due to rather difficult to control a balance between the target tissue (or organ; MGs, in our case), and surrounding tissues (such as the connective tissue of TPs). Also, mRNA microarray experiments are time sensitive as rapid degradation of RNA can occur ex vivo and post mortem. Fortunately, the quality of mRNA samples could be easily controlled by running standard RNA quality checks (and only the samples with RNA Integrity Numbers (RIN) between 7 and 10 were used in our study), while the presence of MGs in (TP + MG) samples was controlled microscopically. Notwithstanding only the semi-quantitative nature of the latter approach, the informativeness of mRNA analyses of (TP + MG) samples was helped by the decidedly low levels of expression of genes of interest in (TP − MG) samples (Figure 6). As a result, GEP of each of the study groups demonstrated highly reproducible, repeatable patterns with no major outliers. The almost identical lipid profiles of male and female samples further corroborated these results.

In conclusion, this first comparative study of meibogenesis in human males and females demonstrated no major differences in their respective lipidomes and transcriptomes, except for expected differences in GEP of known gender-specific genes. Our new data on the levels of expression of major genes related to lipid biosynthesis in general, and meibogenesis specifically, encompassed a group of signature genes of meibogenesis that need to be considered in the future studies of lipid metabolism in ocular tissues and its changes during the onset of various ocular pathologies such as chalazia, Dry Eye, MGD, and MG carcinoma. We believe that our observations provide a hope that treating lipid-related forms of these pathologies could be gender-independent, and could target mechanisms that are common in both sexes.

## 4. Materials and Methods

### 4.1. Collection and Preparation of Study Samples

All study sample collection procedures were approved by the Institutional Review Board of the University of Texas Southwestern Medical Center. The collection procedures were performed in accordance with the principles of the Declaration of Helsinki. Written informed consents were obtained from all study participants. TPs of recently deceased donors (≤ 10 hrs post mortem, obtained through The Willed Body Program, University of Texas Southwestern Medical Center (UTSW)) with no ocular abnormalities and clearly observable MGs (minor MG dropout was allowed, considering the age of the subjects; subjects with moderate to severe MG dropout were disqualified), and surgical samples (in the form of surgery discards) from normal, non-MGD live donors undergoing eye lid corrective surgeries for entropion, ectropion, or skin ulceration (5 females (average age 68 yrs, SD = 13) and 7 males (average age 71 yrs, SD = 9), in total) were collected and immediately processed as described below. The TP tissue samples were dissected free from epidermis and conjunctiva using a dissecting microscope and kept in the RNAlater^®^ solution (from Qiagen, Germantown, MD) at −20 °C until further processing. Samples of meibum were collected from 9 females (average age 64 yrs, SD = 22) and 9 males (average age 62 yrs, SD = 19) essentially as described before [28]. Only samples from subjects with no MGD were used in the study. Note that the subject population was determined by the fact that obtaining specimens from live volunteers was limited only to those who needed eyelid corrective surgeries for other reasons, because no biopsies can be performed on live subjects due to the highly invasive nature of the procedure.

### 4.2. Analytical Procedures

The TP specimens were subjected to RNA extraction and quality check in the UTSW Genomic and Microarray Core Facility. Only the samples that passed initial quality control (i.e., those with RINs between 7 and 10) were used for subsequent transcriptomic analyses. TP specimens were then analyzed using HTA-2_0 mRNA microarrays (from Affymetrix).

Meibum samples were analyzed using chromatographic and mass-spectrometric procedures exactly as described before ([20,21,22] and references cited therein). Briefly, normal phase high performance liquid chromatography on a Diol silica gel column (Lichrosphere 3.2 × 150 mm, 5 μm; from Phenomenex) in combination with ion trap mass spectrometry in positive ion mode [28,40] was used to conduct preliminary evaluation of the samples. Then, the samples were analyzed using Synapt G2-Si high resolution qTOF mass spectrometer equipped with an IonSabre atmospheric pressure chemical ionization ion source and an M-Class UPLC system operated in the reverse phase mode (both instruments were from Waters Corp., Milford, MA). An Acquity UPLC C_18_ BEH column (1 × 100 mm, 1.7 μm) was used to separate the analytes in a binary *i*-propanol/acetonitrile gradient as described earlier [21,22]. Reproducibility of the analyses was evaluated using multiple injections of the same sample (0.5 μL in *i*-propanol), while linearity of the instrument’s response—by injecting increasing volumes of the sample solution (between 0.1 and 1.0 μL).

### 4.3. Data Analyses

The transcriptomic datasets were processed using Expression and Transcriptome Analysis Consoles (v.4.0.1.36; both from Affymetrix) and SigmaStat (v.3.5, from Systat Software, Inc., San Jose, CA, USA). The default (and currently the industry standard) filter criteria: (1) (+2) < LFC < (−2), and (2) ANOVA *p*-value (condition pair) ≤0.05, were used to analyze the data. A tighter LFC of >(+1.2) and <(−1.2), as proposed in [34], was also tested, but deemed impractical because of an unrealistically high number of samples needed to satisfy statistical criteria (see Discussion).

The RP-UPLC/MS data were analyzed using MassLynx (v.4.1), MSe Data Viewer (v.1.4), and Progenesis QI software packages (from Waters). A Supplemental Table S1 lists major lipids of human meibum relevant to this study, and their corresponding *m/z* values. SigmaStat and SigmaPlot software packages from Systat Software, Inc. were used to conduct statistical evaluation of the data.

The transcriptomic and lipidomic data for two genders were compared gender-wide using Student’s *t*-test for the two groups. Tests with *p*-values ≤ 0.05 were considered statistically significant. Principal component analyses were performed using Transcriptome Analysis Console, Progenesis QI, and EZInfo (v.3.0.3.0 from Umetrics AB, Umeå, Sweden).

## 5. Conclusions

Taken together, our data suggest that in humans of both genders meibogenesis proceeds in a similar fashion and produces meibomian gland secretions with virtually identical, and highly conserved, compositions due to, in part, virtually identical expression levels of signature genes of meibogenesis in all study groups. This reinforces the idea about a critical role of meibum in ocular surface health, as the human body, in normal conditions, does not allow large variations in the meibomian lipidome. Our findings may contribute to the development of novel, gender-independent diagnostic tools and therapeutic methods for treating various MG-related diseases and pathological conditions.

## Figures and Tables

**Table 1:** Targeted transcriptomic analysis of human tarsal plates. Genes of meibogenesis.

Gene	Females, Mean Bi-Weight Signal, Log2	Females, Standard Deviation, Log2	Males, Mean Bi-Weight Signal, Log2	Males, Standard Deviation, Log2	Significance, *p* (α = 0.05)
*AADACL4*	13.48	1.69	13.82	0.72	0.690
*ABHD5*	12.94	1.44	12.49	0.67	0.544
*ACADVL*	10.06	0.73	11.59	0.18	0.002 *
*ACAT2*	15.47	1.31	15.05	1.13	0.602
*ACSL1*	13.3	1.11	13.24	0.7	0.921
*ACSL3*	16.27	1.03	16.24	0.75	0.959
*ACSL5*	11.56	1.13	11.65	0.94	0.894
*APOD*	13.24	1.29	14.24	0.6	0.155
*AWAT1*	15.01	1.96	13.28	1.46	0.152
*AWAT2*	16.7	1.07	16.54	0.55	0.774
*CES1*	13.35	0.89	14.39	1.64	0.48
*CYP4F22*	13.38	1.12	12.97	0.6	0.491
*CYP4X1*	12.2	0.45	13.42	0.94	0.031 *
*CYP51A1*	13.42	1.33	13.15	1.11	0.736
*DBT*	11.18	0.81	11.25	0.79	0.893
*DGAT2*	13.96	1.28	13.01	1.17	0.255
*DHCR24*	17.26	0.66	16.79	0.67	0.296
*DHCR7*	14.7	1.14	13.54	0.73	0.092
*ECHDC1*	12.53	0.81	12.2	0.58	0.480
*EGFR*	12.27	0.38	12.4	0.67	0.716
*ELOVL1*	11.98	1.07	11.74	0.75	0.692
*ELOVL2*	5.68	0.76	5.18	0.29	0.207
*ELOVL3*	14.58	1.11	14.79	1	0.761
*ELOVL4*	18.71	0.9	17.62	0.86	0.086
*ELOVL5*	9.79	1.15	9.21	0.62	0.35
*ELOVL6*	14.41	0.85	14.36	1	0.934
*ELOVL7*	9.04	0.75	8.55	0.38	0.229
*FAR2*	18.02	0.83	17.08	0.81	0.107
*FASN*	11.86	1	11.07	0.52	0.156
*GPAM*	11.15	1.11	10.61	0.73	0.390
*HMGCR*	17.12	1.34	16.92	0.84	0.785
*HMGCS1*	16.79	0.75	16.47	0.95	0.571
*HSD11B1*	11.45	1.45	11.46	1.06	0.99
*HSD17B12*	13.85	0.63	13.04	1.24	0.229
*HSD17B2*	15.41	1.15	15.36	0.5	0.931
*HSD17B4*	11.65	0.29	11.97	0.22	0.085
*HSDL2*	12.46	0.51	12.15	0.6	0.404
*IFITM2*	14.12	1.75	12.33	0.63	0.064
*MSMO1*	16.61	0.8	16.23	0.98	0.521
*PEBP1*	13.75	0.7	13.77	0.31	0.955
*SCARB2*	11.36	0.7	11.43	0.38	0.849
*SCD*	19.41	0.4	19.24	0.39	0.515
*SLC12A2*	13.97	2.01	12.62	1.46	0.259
*SLC25A6*	13.44	0.33	13.29	0.48	0.581
*SLC31A1*	14.46	1.36	13.03	0.54	0.06
*SLC38A2*	14.95	0.71	15.34	0.56	0.363
*SLCO4C1*	13.87	1.41	11.77	0.97	0.025 *
*SOAT1*	17.2	0.75	17.47	0.9	0.620
*SOAT2*	5.13	0.25	4.90	0.39	0.299
*SQLE*	13.49	1.4	12.9	0.95	0.458

Top 50 expressed protein-coding genes detected in (TP + MG) tarsal plate samples of both genders using targeted transcriptomic analysis (*n* = 10; 5 males and 5 females; averaged data). Statistically significant differences are shown in red and labeled with an asterisk (*).

## Supplementary Materials

Supplementary materials can be found at https://www.mdpi.com/1422-0067/20/18/4539/s1.

## Abbreviations

AP	Atmospheric pressure chemical ionization
CE	Cholesteryl ester(s)
Ch	Free cholesterol
Chl-OAHFA	Cholesteryl ester(s) of (*O*)-acylated omega-hydroxy fatty acid(s)
CT	Connective tissue
DiAD	Diacylated diol(s)
ELCFA	Extremely long chain fatty acid(s)
FA	Fatty acid(s)
FAl	Fatty alcohol(s)
GEP	Gene expression profile(s)
LFC	Linear fold change
MG	Meibomian gland(s)
MGD	Meibomian gland dysfunction
MS	Mass spectrometry
NIM	Negative ion mode
OAHFA	(*O*)-acylated omega-hydroxy fatty acid(s)
PCA	Principal component analysis
PIM	positive ion mode
RP	Reversed phase
RT	Retention time(s)
TAG	Triacylglycerol(s)
TIC	Total ion chromatogram(s)
TOF	Time-of-Flight
TP	Tarsal plate(s)
UPLC	Ultra-high performance liquid chromatography
VLCFA	Very long chain fatty acid(s)
WE	Wax ester(s)
