# Supplementary material for: Comparative Transcriptomic and Lipidomic Analyses of Human Male and Female Meibomian Glands Reveal Common Signature Genes of Meibogenesis
Source: Int J Mol Sci. 2019 Sep 13;20(18):4539. doi: 10.3390/ijms20184539 (PMC6769918; doi:10.3390/ijms20184539)
Supplement: Supplementary file 1 [file ijms-20-04539-s001.pdf]

Supplemental Table 1. Major classes of lipids of human meibum detectable in RP-UPLC/MS experiments in positive and negative ion modes.

**Saturated wax esters**	**Theoretical *m/z*, (M + H)$^+$**	**Mono-unsaturated wax esters**	**Theoretical *m/z*, (M + H)$^+$**
$C_{36}H_{72}O_2$	537.56	$C_{36}H_{70}O_2$	535.55
$C_{37}H_{74}O_2$	551.58	$C_{37}H_{72}O_2$	549.56
$C_{38}H_{76}O_2$	565.59	$C_{38}H_{74}O_2$	563.58
$C_{39}H_{78}O_2$	579.61	$C_{39}H_{76}O_2$	577.59
$C_{40}H_{80}O_2$	593.62	$C_{40}H_{78}O_2$	591.61
$C_{41}H_{82}O_2$	607.64	$C_{41}H_{80}O_2$	605.62
$C_{42}H_{84}O_2$	621.65	$C_{42}H_{82}O_2$	619.64
$C_{43}H_{86}O_2$	635.67	$C_{43}H_{84}O_2$	633.65
$C_{44}H_{88}O_2$	649.69	$C_{44}H_{86}O_2$	647.66
$C_{45}H_{90}O_2$	663.70	$C_{45}H_{88}O_2$	661.69
$C_{46}H_{92}O_2$	677.72	$C_{46}H_{90}O_2$	675.70
$C_{47}H_{94}O_2$	691.73	$C_{47}H_{92}O_2$	689.72
$C_{48}H_{96}O_2$	705.5	$C_{48}H_{94}O_2$	703.73
$C_{49}H_{98}O_2$	719.76	$C_{49}H_{96}O_2$	717.75
$C_{50}H_{100}O_2$	733.78	$C_{50}H_{98}O_2$	731.76
**Saturated cholesteryl esters**	**Theoretical m/z, (M – 369 + H)$^+$**	**Mono-unsaturated cholesteryl esters**	**Theoretical m/z, (M + H)$^+$**
$C_{14:0}$-CE	597.56	$C_{14:1}$-CE	595.55
$C_{15:0}$-CE	611.58	$C_{15:1}$-CE	609.56
$C_{16:0}$-CE	625.59	$C_{16:1}$-CE	623.58
$C_{17:0}$-CE	639.61	$C_{17:1}$-CE	637.59
$C_{18:0}$-CE	653.62	$C_{18:1}$-CE	651.61
$C_{19:0}$-CE	667.64	$C_{19:1}$-CE	665.62
$C_{20:0}$-CE	681.65	$C_{20:1}$-CE	679.64
$C_{21:0}$-CE	695.67	$C_{21:1}$-CE	693.65
$C_{22:0}$-CE	709.69	$C_{22:1}$-CE	707.67
$C_{23:0}$-CE	723.70	$C_{23:1}$-CE	721.69
$C_{24:0}$-CE	737.72	$C_{24:1}$-CE	735.70
$C_{25:0}$-CE	751.73	$C_{25:1}$-CE	749.72
$C_{26:0}$-CE	765.75	$C_{26:1}$-CE	763.73
$C_{27:0}$-CE	779.76	$C_{27:1}$-CE	777.75
$C_{28:0}$-CE	793.78	$C_{28:1}$-CE	791.76
$C_{29:0}$-CE	807.80	$C_{29:1}$-CE	805.78
$C_{30:0}$-CE	821.81	$C_{30:1}$-CE	819.79
$C_{31:0}$-CE	835.83	$C_{31:1}$-CE	833.81
$C_{32:0}$-CE	849.84	$C_{32:1}$-CE	847.83
$C_{33:0}$-CE	863.86	$C_{33:1}$-CE	861.84
$C_{34:0}$-CE	877.87	$C_{34:1}$-CE	875.86
$C_{35:0}$-CE	891.89	$C_{35:1}$-CE	889.87

Mono-unsaturated Chl-OAHFA	Theoretical m/z, $(M + H)^+$	Diunsaturated Chl-OAHFA,	Theoretical m/z, $(M + H)^+$
$C_{65}H_{116}O_4$	961.89	$C_{65}H_{114}O_4$	959.88
$C_{66}H_{118}O_4$	975.91	$C_{66}H_{116}O_4$	973.89
$C_{67}H_{120}O_4$	989.93	$C_{67}H_{118}O_4$	987.91
$C_{68}H_{122}O_4$	1003.94	$C_{68}H_{120}O_4$	1001.93
$C_{69}H_{124}O_4$	1017.96	$C_{69}H_{122}O_4$	1015.94
$C_{70}H_{126}O_4$	1031.97	$C_{70}H_{124}O_4$	1029.96
$C_{71}H_{128}O_4$	1045.99	$C_{71}H_{126}O_4$	1043.97
$C_{72}H_{130}O_4$	1060.00	$C_{72}H_{128}O_4$	1057.99
$C_{73}H_{132}O_4$	1074.02	$C_{73}H_{130}O_4$	1072.00
$C_{74}H_{134}O_4$	1088.03	$C_{74}H_{132}O_4$	1086.02
$C_{75}H_{136}O_4$	1102.05	$C_{75}H_{134}O_4$	1100.03
$C_{76}H_{138}O_4$	1116.07	$C_{76}H_{136}O_4$	1114.05
$C_{77}H_{140}O_4$	1130.08	$C_{77}H_{138}O_4$	1128.07
$C_{78}H_{142}O_4$	1144.10	$C_{78}H_{140}O_4$	1142.08
$C_{79}H_{144}O_4$	1158.11	$C_{79}H_{142}O_4$	1156.10
$C_{80}H_{146}O_4$	1172.13	$C_{80}H_{144}O_4$	1170.11
**Triacylglycerols**	**Theoretical m/z, $(M + H)^+$**	**Triacylglycerols**	**Theoretical m/z, $(M + H)^+$**
$C_{51}H_{93}O_6$	801.697	$C_{55}H_{101}O_6$	857.759
$C_{51}H_{95}O_6$	803.708	$C_{55}H_{103}O_6$	859.772
$C_{51}H_{97}O_6$	805.727	$C_{57}H_{101}O_6$	881.759
$C_{53}H_{95}O_6$	827.712	$C_{57}H_{103}O_6$	883.775
$C_{53}H_{97}O_6$	829.725	$C_{57}H_{105}O_6$	885.791
$C_{53}H_{99}O_6$	831.744	$C_{59}H_{107}O_6$	911.806
$C_{55}H_{97}O_6$	853.728	$C_{60}H_{107}O_6$	923.806
$C_{55}H_{99}O_6$	855.744		
**OAHFA**	**Theoretical m/z, $(M - H)^-$**	**OAHFA**	**Theoretical m/z, $(M - H)^-$**
$C_{48}H_{89}O_4$	729.677	$C_{50}H_{93}O_4$	757.707
$C_{52}H_{97}O_4$	785.739		
